# Supplementary material for: Comparative Transcriptome Analysis Reveals the Effect of Aurantiochytrium sp. on Gonadal Development in Zebrafish
Source: Animals (Basel). 2023 Aug 1;13(15):2482. doi: 10.3390/ani13152482 (PMC10417364; doi:10.3390/ani13152482)
Supplement: Supplementary file 1 [file animals-13-02482-s001.zip › animals-2485430-supplementary.pdf]

Table S1 *Aurantiochytrium* Extract Main Ingredients

Major fatty acids	Content
Methyl tetradecanoate	2.34%
Pentadecanoic acid, methyl ester	2.44%
Hexadecanoic acid, methyl ester	30.21%
Methyl4, 7, 10, 13, 16-docosapentaenoate	9.07%
4, 7, 10, 13, 16, 19-docosahexaenoic acid, methylester	50.19%

Table S2 Primers Used for Detection of DEGs

Gene	Primer name	Primer sequence (5′ -3′)	Purpose	Accession No.
acsl2	*acsl2*-F	GGACAGAGGGAGGAAGTGCG	RT-qPCR	ENSDARG00000078399
	acsl2-R	TGACCGAGGAGGTGTTGGAG	RT-qPCR	
apoc2	*apoc2*-F	CCCTGAAATCCTACTATGACC	RT-qPCR	ENSDARG00000092155
	apoc2-R	TGAAGACAAGAGTTCGCCACT	RT-qPCR	
apoeb	*apoeb* -F	AAACGCCTGAACAAGGACAC	RT-qPCR	ENSDARG00000040295
	apoeb -R	TGCGTAGGTTCTCGGCTGTC	RT-qPCR	
blocls3	*blocls*3-F	TCCCTGCGTCATTATCCTCC	RT-qPCR	ENSDARG00000097344
	blocls3-R	GCATCTGCTGTTGGCTTTCA	RT-qPCR	
cel.1	*cel.1*-F	TCTCGTATTCCTGTCTTCCCTCT	RT-qPCR	ENSDARG00000017490
	cel.1-R	ACCTTGGACTCGCCTTTGTTAG	RT-qPCR	
cel.2	*cel.2*-F	CAGGGAAAGAGTCATAGTGTTGG	RT-qPCR	ENSDARG00000029822
	cel.2-R	ATCCGTTTGAAGGAGATTGAGC	RT-qPCR	
fabp4a	*fabp4a* -F	GGGCTGATCTGCATGAAGTCT	RT-qPCR	ENSDARG00000017299
	fabp4a -R	AGTTTGCCGTTCTCGATAGTC	RT-qPCR	
gck	*gck* -F	GAAGGCTCCGAGGTTGGTGATT	RT-qPCR	ENSDARG00000068006
	gck -R	CGGTCATGGCATCTTCAGGGAT	RT-qPCR	
lox	*lox*-F	AGGCTGGACTCGCTTGTATT	RT-qPCR	ENSDARG00000016789
	lox-R	CCGTCTGAAAGGATTGTGGG	RT-qPCR	
pllp	*pllp* -F	TGAGTCTTCATCAGCGGTTAC	RT-qPCR	ENSDARG00000062756
	pllp -R	AGCCCAAATAGGCAAAGTAGC	RT-qPCR	
sqlea	*sqlea* -F	CTGCCCTCGCTGAGCCTAAT	RT-qPCR	ENSDARG00000079946
	sqlea -R	CGAACCTCCTTGAGCATCCC	RT-qPCR	
tnfrsf1b	*tnfrsf1b*-F	ACCGAATGTGAAGGTAGTGC	RT-qPCR	ENSDARG00000070165
	tnfrsf1b -R	TCCCTTGTGCGTTTGTCGT	RT-qPCR	
vtg1	*vtg1*-F	CAGCTTGTTTTGGACAGGGC	RT-qPCR	ENSDARG00000078429
	vtg1-R	GATGAGAGCCACTGAAGGCT	RT-qPCR	
vtg2	*vtg2*-F	TTGAAACTACGGGAAATCTTG	RT-qPCR	ENSDARG00000103451
	vtg2-R	GCTACTGGTACTGTTGCTGGT	RT-qPCR	
vtg3	*vtg3*-F	GCCACTTGAGTTACCGCTACA	RT-qPCR	ENSDARG00000016448
	vtg3-R	AGTCCATCTGCACGGATACCA	RT-qPCR	
vtg7	*vtg7*-F	TTGAGCAAACGCAGAAACAGAA	RT-qPCR	ENSDARG00000092419

	vtg7-R	AGTAAGCAGCGAGTTGGTAGCC	RT-qPCR	
inhbb	*inhbb*-F	CAGTTTTGCCGAATCAGATGATGTA	RT-qPCR	ENSDARG00000040777
	Inhbb-R	TAGGAGTGCACCCTCACTGT	RT-qPCR	
egf	*egf*-F	AGCCACTCCAGAGGTCTATGT	RT-qPCR	ENSDARG00000052739
	egf -R	CAACAGCCAGGCTACAACCA	RT-qPCR	
igf3	*igf3*-F	GCCAAACGCCTTCAGATAAT	RT-qPCR	ENSDARG00000058058
	igf3-R	CTACAATGCCTTTCCCACGA	RT-qPCR	
esr1	*esr1*-F	ACTCTCACCCATGTACCCTAAGG	RT-qPCR	ENSDARG00000004111
	esr1-R	CGGGTAGTATCCCACTGAAGC	RT-qPCR	
fgf24	*fgf24*-F	CTGTTCTGCCGTCAAGGTTCA	RT-qPCR	ENSDARG00000037677
	fgf24-R	CCATCGTCTCCATTGGCGTTA	RT-qPCR	
β-actin	*β-actin*-F	GGCATCACACCTTCTACAA	Reference gene	NM_131031.2
	β-actin-R	CAGAGTCCATCACAATACCA	Reference gene	

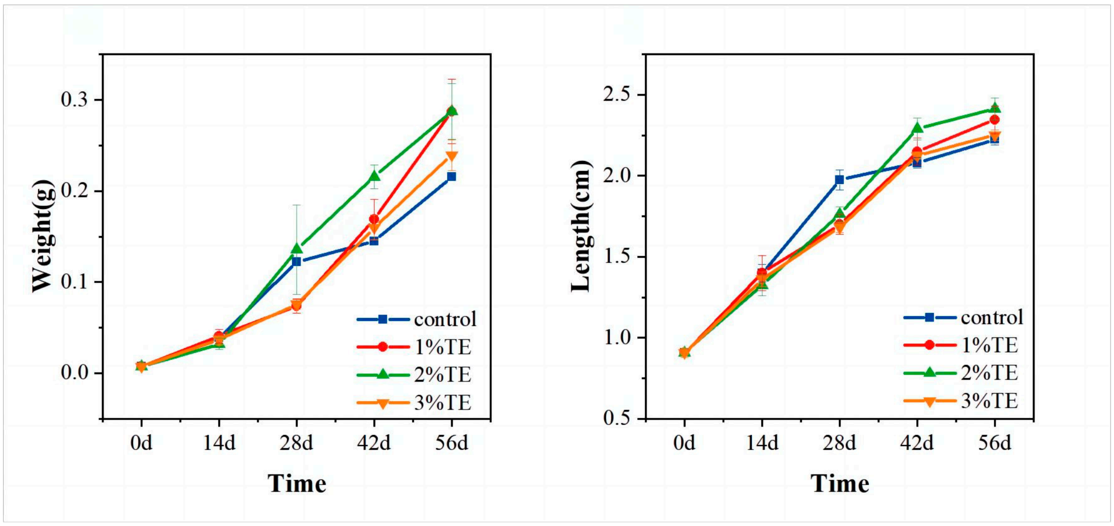

Figure S1 Variation in body weight and length in zebrafish
